# Supplementary material for: Topological and kinetic determinants of the modal matrices of dynamic models of metabolism
Source: PLoS One. 2017 Dec 21;12(12):e0189880. doi: 10.1371/journal.pone.0189880 (PMC5739448; doi:10.1371/journal.pone.0189880)
Supplement: S1 Text — (PDF) [file pone.0189880.s001.pdf]

Supporting text

Topological and kinetic determinants of the modal matrices of dynamic models of metabolism

Bin Du[¶], Daniel C. Zielinski[¶], Bernhard O. Palsson*

Department of Bioengineering, University of California, San Diego, La Jolla, California, United States of America

*Corresponding author: Bernhard O. Palsson
E-mail: palsson@ucsd.edu

[¶]These authors contributed equally to this work.

Properties of modes of the Jacobian matrix in a metabolic network

As described in the main text, the eigenvalues and modes (left eigenvectors) of the Jacobian matrix give information on the system dynamics around a steady state. Each mode is associated with a particular eigenvalue and represents the dynamic motion in a characteristic time scale defined by the eigenvalue. Such characteristic time scales describe the approximate time it takes for the mode to relax (return to the original state) following a perturbation. When the metabolic network is perturbed from the steady state, the modes respond in order of their characteristic time scales. We label each mode with a number i based on their response time, from fast modes to slow modes. The dynamic response of a concentration mode i can be expressed in the form of $m_i(t) = m_{i0} \cdot e^{\lambda_i t}$, where λ_i is the corresponding eigenvalue and m_{i0} is the inner product of left eigenvector u_i and steady state concentration vector $\mathbf{x}$ ($\mathbf{v}$ in the case of flux mode) at time 0. The coefficient in left eigenvector u_i represents the contribution of element response to the mode response. As the effect of perturbation propagates through time, slower modes get excited and faster modes relax. When a mode relaxes, we have $m_i = 0$. The direction at which the mode moves depends on the elements in the mode and the elements in the perturbation vector. For example, when the perturbation is the change of ATP concentration, the first concentration mode to respond must have a non-zero ATP coefficient in that left eigenvector.

Diagonal dominance and the Gershgorin circle theorem

Gershgorin circle theorem can help explain eigenvalues in cases where diagonal dominance occurs in the Jacobian matrix. In this theorem, each row of the Jacobian matrix forms a Gershgorin circle, with the diagonal element $\mathbf{J}_{ii}$ being the center and the absolute sum of off-diagonal elements $\sum_{k \neq i} |\mathbf{J}_{ik}|$ being the radius. The Gershgorin circle theorem constrains the eigenvalues based on the relative magnitudes of diagonal and off-diagonal elements in a matrix. As illustrated in Fig 2B, the theorem is helpful in associating eigenvalues with certain

Gershgorin circles, particularly when the row is strongly diagonally dominant, where the diagonal element is much greater than the sum of off-diagonal elements in absolute value.

However, as the diagonal dominance gets weaker, the radius of the circle becomes larger and it becomes difficult to associate a unique eigenvalue to each circle. In the case where the row is not diagonally dominant, the circle can reach the positive part of the x-axis and possibly contain positive eigenvalues when the system is unstable. In our system, we did not find any positive eigenvalues. It is also worth mentioning that eigenvalues will fall in any Gershgorin circles but not all Gershgorin circles will contain an eigenvalue, especially in cases where the circles overlap with each other. However, even though overlapping occurs frequently in our model, we found that 99.0% of the circles had an eigenvalue inside, which may not be guaranteed for all models.

Negative diagonal elements in the Jacobian matrix encourage system stability

During the course of analysis of diagonal dominance and eigenvalues of the Jacobian matrix, we noticed a critical relationship between diagonal dominance in metabolic networks and system stability. The local stability of a fixed point (steady-state) of a dynamic system is determined by the presence of only negative eigenvalues of the Jacobian matrix. We note that the diagonal elements are always negative in the Jacobian matrix of a biochemical network, due to the formulation of mass action rate law. Because of this, the eigenvalues that fall in the Gershgorin circles formed from diagonally dominant rows are guaranteed to be negative, ensuring the stability of the system. On the other hand, when the row is not diagonally dominant, the Gershgorin circle crosses 0 on the x-axis, enabling positive eigenvalues to appear.

Examining the impact of the negative diagonal elements on system stability, we replaced the diagonal elements of **J** with zero value one at a time and recalculated the eigenvalues of **J**. This effectively moves center of the related Gershgorin circle to the origin, while the radius is unchanged as it is set by the off-diagonal elements. We found that there are always positive eigenvalues after modifying the diagonal element and the largest positive eigenvalue tends to be close to the replaced diagonal element in absolute value (S2 Fig). Since the Gershgorin circle surrounds the origin, it would seem that the resulting eigenvalue could be either positive or negative; however, we did not observe this to be the case. The mathematical origin of this phenomenon is thus not entirely clear to us. Still, we concluded that the negative diagonal elements of the Jacobian matrix are clearly critical for system stability, with the effect magnified by diagonal dominance.

Table A. Examples of diagonally dominant metabolites and their corresponding mode structures in the whole-cell RBC kinetic model

Diagonally dominant metabolite	Diagonal fraction	Characteristic time (hour)	Concentration mode structure
G6PDH&NADP&G6P	0.000306	1.93×10^{-8}	*G6PDH*&NADP&G6P
PFK_T	0.00110	1.10×10^{-11}	*PFK_T*
OAA	0.00167	6.53×10^{-5}	OAA
PPi	0.00169	0.253	PPi
5MDRU1P	0.00262	1.0229	5MDRU1P
GND&NADP&6PGC	0.00308	9.42×10^{-9}	*GND*&NADP&6PGC
GAPDH_T	0.00608	6.04×10^{-11}	*GAPDH_T*
G6PDH&6PGL	0.142	2.45×10^{-9}	*G6PDH*&6PGL,*G6PDH*&NADPH&6PGL

Enzyme forms are in italic. '&' means the enzyme form is bound with metabolite(s). '_T' means that the enzyme is at the tense state. G6P, Glucose 6-phosphate; NADP, Nicotinamide adenine dinucleotide phosphate (oxidized); NADPH, Nicotinamide adenine dinucleotide phosphate (reduced); OAA, Oxaloacetate; PPi, Diphosphate; 5MDRU1P, 5-Methylthio-5-deoxy-D-ribulose 1-phosphate; 6PGC, 6-Phospho-D-gluconate; 6PGL, 6-phospho-D-glucono-1,5-lactone; *GND*, phosphogluconate dehydrogenase; *G6PDH*, glucose 6-phosphate dehydrogenase; *GAPDH*, glyceraldehyde 3-phosphate dehydrogenase; *PFK*, phosphofructokinase; *PGI*, glucose 6-phosphate isomerase.

Table B. Statistics on diagonal dominance in rows of concentration Jacobian matrix of the whole-cell RBC kinetic model

Diagonal fraction range	Percentage in **J** (%)
< 0.1	9.93
0.1 - 1	26.0
1 - 10	39.4
> 10	24.0
