# Supplementary figures and images for: Topological and kinetic determinants of the modal matrices of dynamic models of metabolism
Source: PLoS One. 2017 Dec 21;12(12):e0189880. doi: 10.1371/journal.pone.0189880 (PMC5739448; doi:10.1371/journal.pone.0189880)

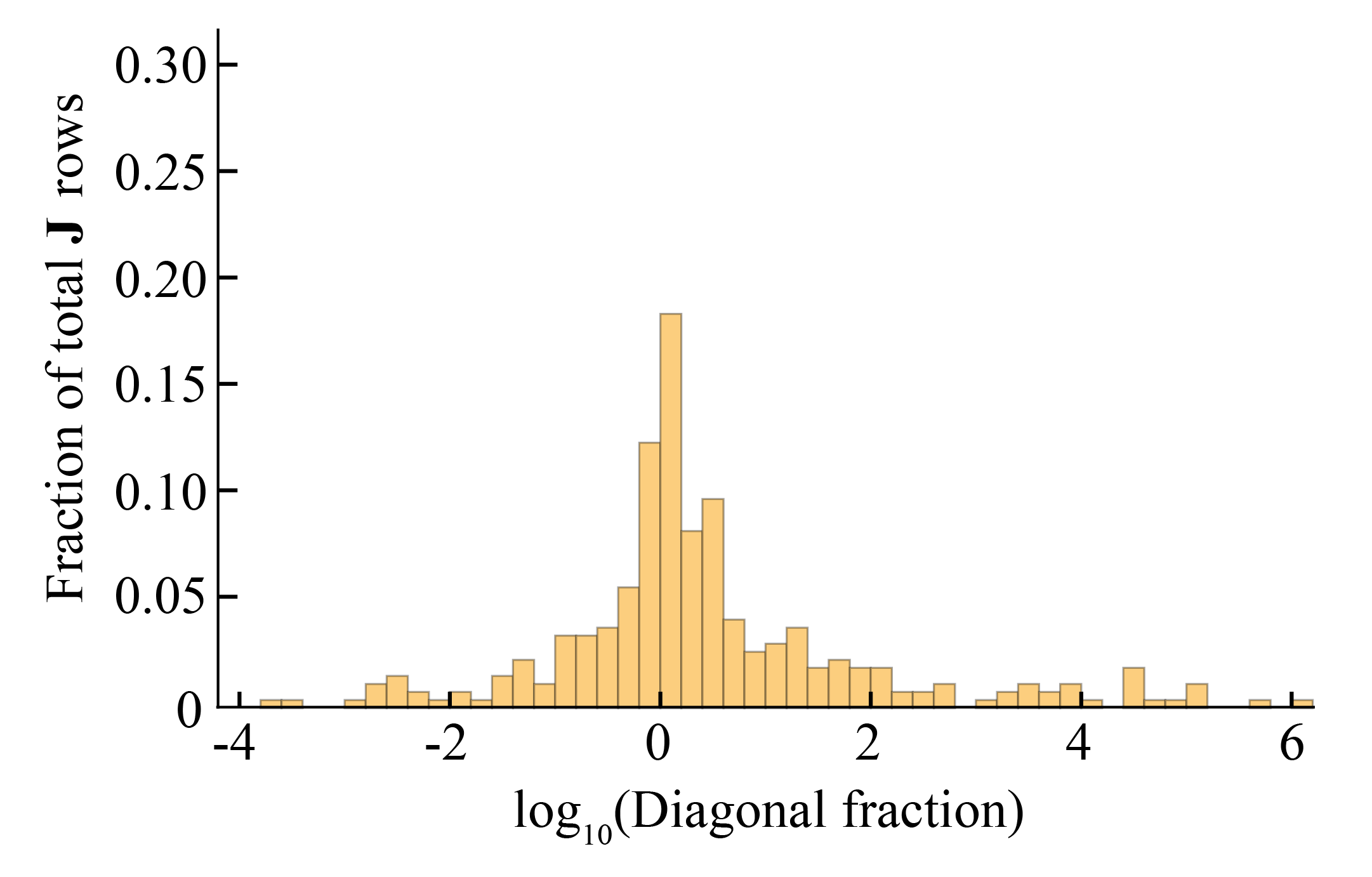

Supplement: S1 Fig — The distribution of fraction of total J rows in terms of diagonal fraction on a log10 scale. (TIF) [file pone.0189880.s003.tif]

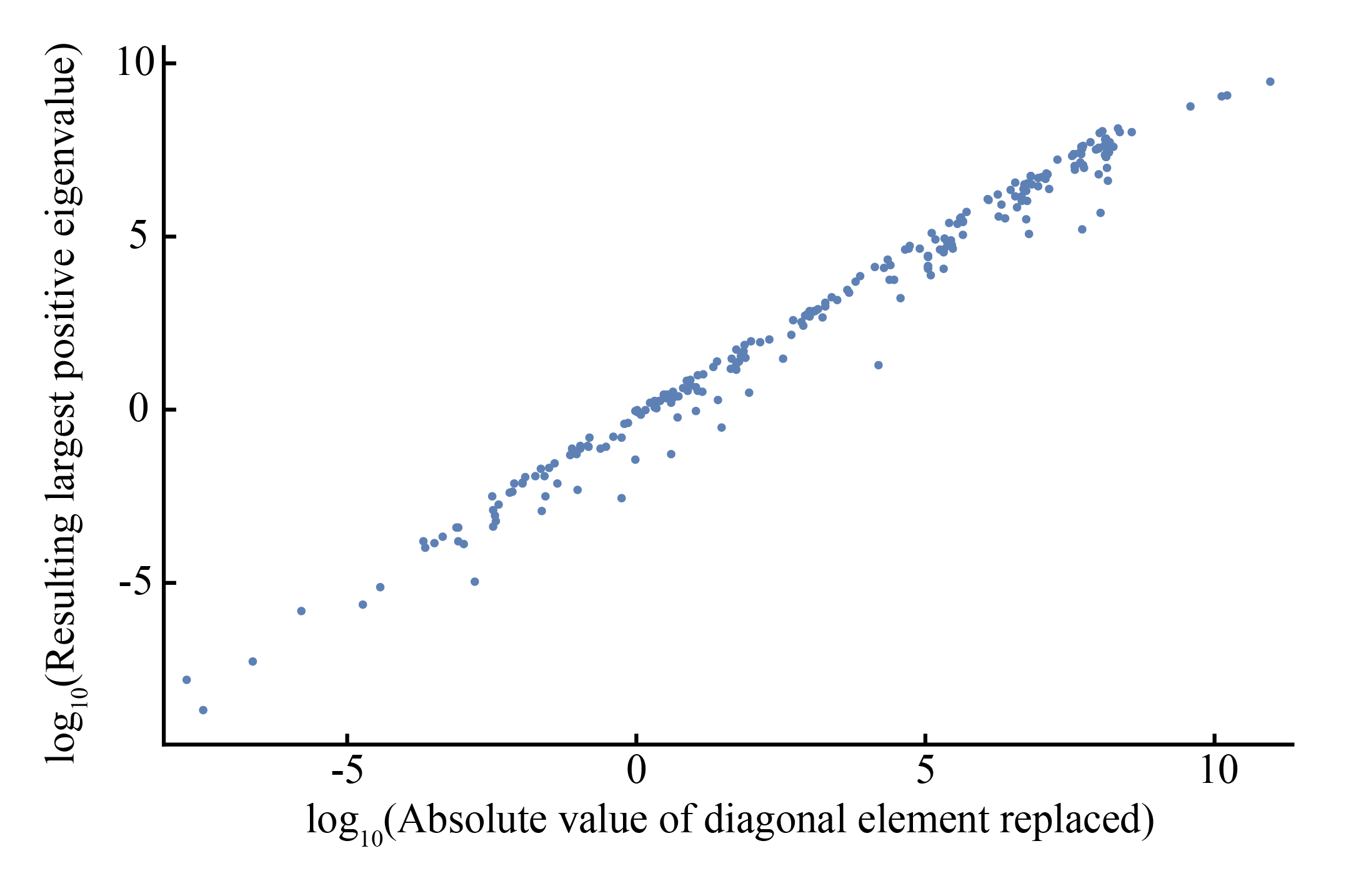

Supplement: S2 Fig — We replaced each diagonal element of the Jacobian matrix with zero value one at a time and calculated the eigenvalues of the modified matrix. We observed that the largest positive eigenvalues are on the same order of magnitude as the absolute values of the diagonal elements replaced. (TIF) [file pone.0189880.s004.tif]

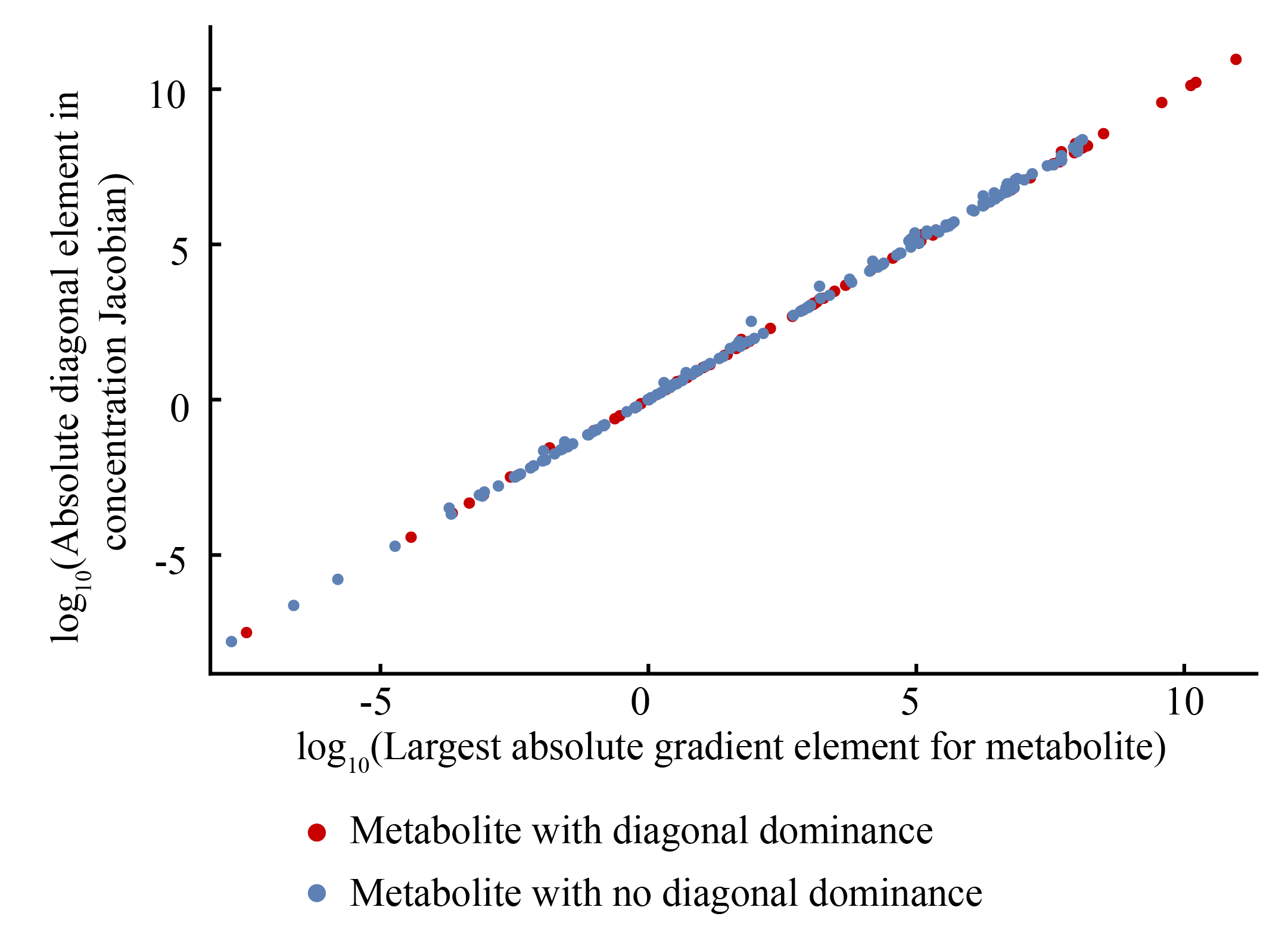

Supplement: S3 Fig — Metabolites with diagonal dominance are marked red while metabolites with no diagonal dominance are marked blue. The diagonal element of J is largely determined by a single value within G, suggesting that diagonal dominance can be tied to a single reaction sensitivity (dv/dx) in each case. (TIF) [file pone.0189880.s005.tif]

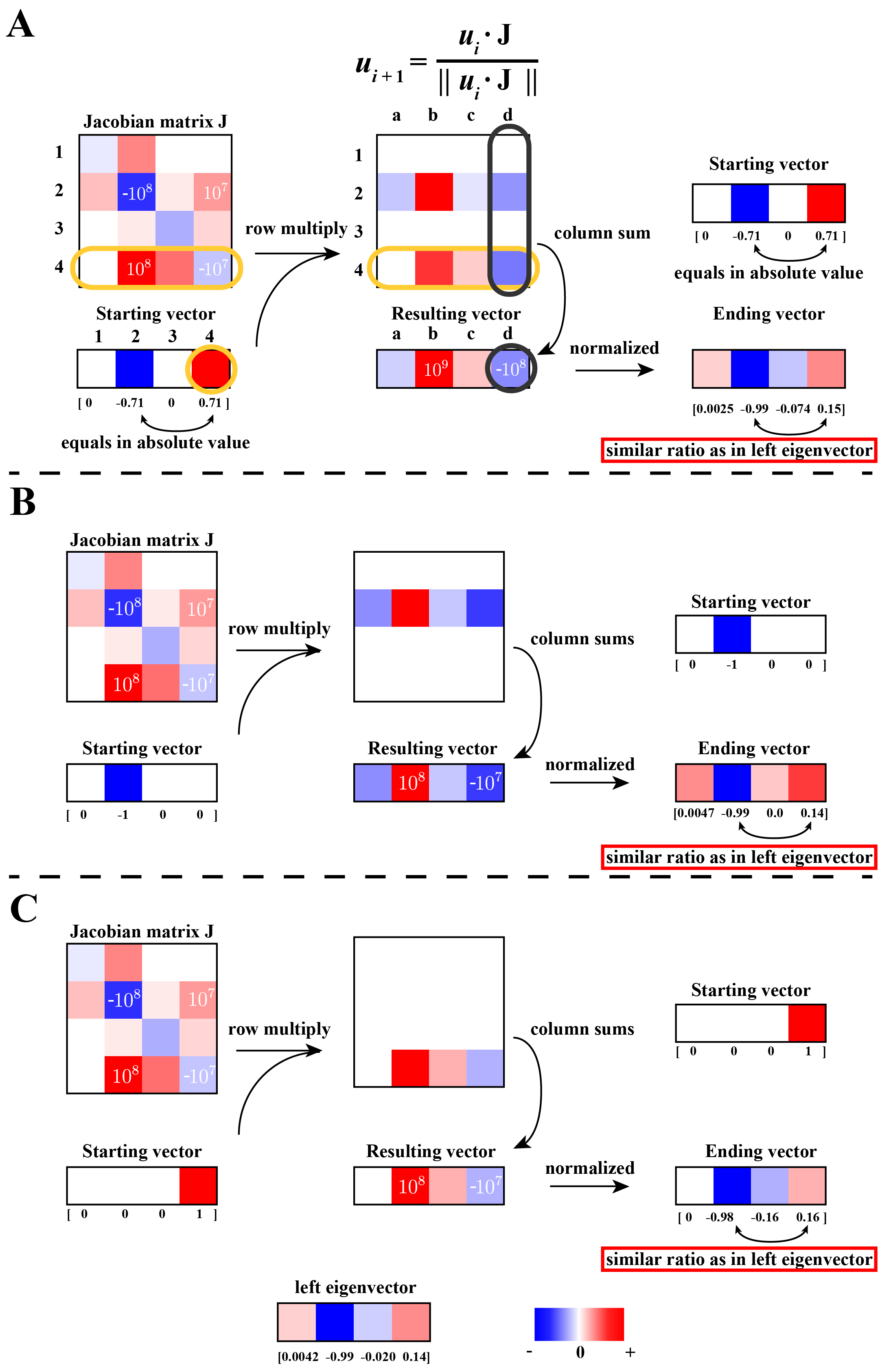

Supplement: S4 Fig — We divide the vector multiplication with the Jacobian matrix into multiple steps. First of all, each row of the Jacobian matrix is multiplied by every element of the starting vector (Panel A yellow circles). We then sum up each column of the second matrix to obtain the resulting vector (Panel A black circles), which is normalized to give the ending vector. (A) Starting vector with nonzero entries at the 2nd and 4th positions multiplied with the Jacobian matrix. The ending vector is very similar to the original eigenvector. This is the same example as in Fig 3B. (B) Starting vector with nonzero entry at the 2nd position multiplied with the Jacobian matrix. We picked this vector to demonstrate its interaction with the 2nd row specifically. The ending vector is very similar to the actual eigenvector, showing that the 2nd row is one of the determining factors for the eigenvector. (C) Starting vector with nonzero entry at the 4th position multiplied with the Jacobian matrix. Similar to the previous example, we picked this vector to demonstrate its interaction with the 4th row. The ending vector is very similar to the actual eigenvector, showing that the 4th row also contributes to eigenvector formation. Overall, the examples above demonstrate that both the 2nd and 4th rows contribute to the structure of the eigenvector similarly. The Jacobian matrix presented here corresponds to G6PDH enzyme forms and is a submatrix of J from the RBC metabolic network. The large values in the Jacobian submatrix come from the large rate constants of G6PDH enzymatic reactions. For clear demonstration purposes, the comparison of relative colors only works for individual box (surrounded by black stroke) itself, but not across different boxes. (TIF) [file pone.0189880.s006.tif]

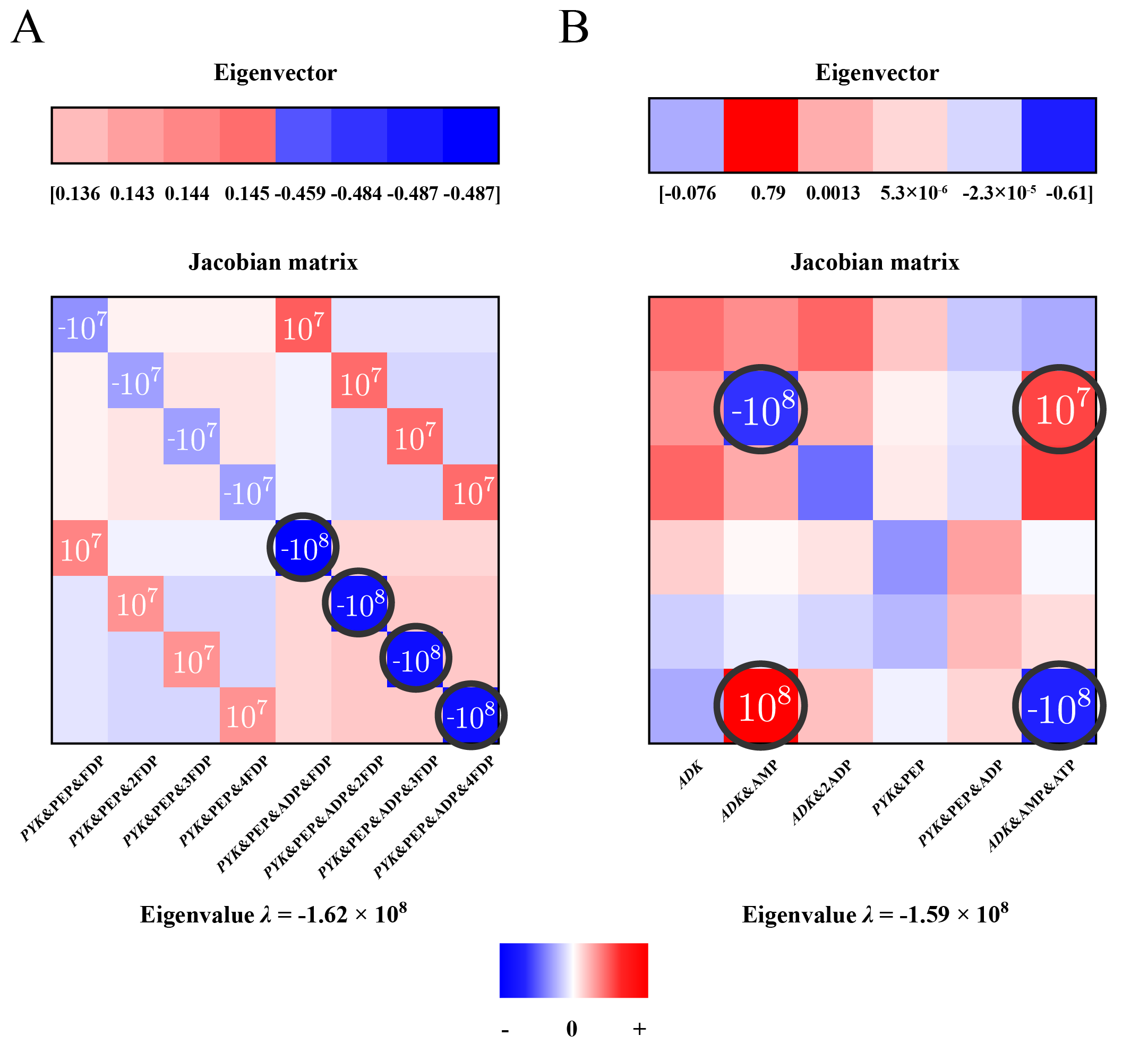

Supplement: S5 Fig — We identified more cases in which complicated mode structures are determined from topologically connected elements of Jacobian matrix at similar magnitude. Elements that are key in determining the eigenvector structure are circled in black. (A) Mode structure for PYK enzyme forms and its related submatrix of J. The 5th, 6th, 7th, 8th are significant elements of the eigenvector. The Jacobian elements determining such eigenvector structure are found at the diagonal positions (5, 5), (6, 6), (7, 7), (8, 8). (B) Mode structure for ADK and PYK enzyme forms and its related submatrix of J. The 2nd and 6th elements are significant in the eigenvector. Key Jacobian elements affecting the eigenvector structure are located at positions (2, 2), (2, 6), (6, 2), (6, 6). For clear demonstration purposes, the comparison of relative colors only works for individual box (surrounded by black stroke) itself, but not across different boxes. (TIF) [file pone.0189880.s007.tif]
